# Oxygen tension modulates the effects of TNFα in compressed chondrocytes

**DOI:** 10.1007/s00011-016-0991-5

**Published:** 2016-09-22

**Authors:** R. K. Tilwani, S. Vessillier, B. Pingguan-Murphy, D. A. Lee, D. L. Bader, T. T. Chowdhury

**Affiliations:** 1 Institute of Bioengineering, School of Engineering and Materials Science, Queen Mary University of London, Mile End Road, London, E1 4NS UK; 2 Biotherapeutics Group, National Institute for Biological Standards and Control, South Mimms, Potters Bar, Hertfordshire, EN6 3QG UK; 3 Department of Biomedical Engineering, Faculty of Engineering, University of Malaya, Kuala Lumpur, 50603 Malaysia; 4 Faculty of Health Sciences, Southampton General Hospital, University of Southampton, Southampton, SO16 6YD UK

**Keywords:** TNF-α, Oxygen tension, Chondrocyte, Osteoarthritis, Mechanotransduction

## Abstract

**Objective and design:**

Oxygen tension and biomechanical signals are factors that regulate inflammatory mechanisms in chondrocytes. We examined whether low oxygen tension influenced the cells response to TNFα and dynamic compression.

**Materials and methods:**

Chondrocyte/agarose constructs were treated with varying concentrations of TNFα (0.1–100 ng/ml) and cultured at 5 and 21 % oxygen tension for 48 h. In separate experiments, constructs were subjected to dynamic compression (15 %) and treated with TNFα (10 ng/ml) and/or L-NIO (1 mM) at 5 and 21 % oxygen tension using an ex vivo bioreactor for 48 h. Markers for catabolic activity (NO, PGE_2_) and tissue remodelling (GAG, MMPs) were quantified by biochemical assay. ADAMTS-5 and MMP-13 expression were examined by real-time qPCR. 2-way ANOVA and a post hoc Bonferroni-corrected *t* test were used to analyse data.

**Results:**

TNFα dose-dependently increased NO, PGE_2_ and MMP activity (all *p* < 0.001) and induced MMP-13 (*p* < 0.05) and ADAMTS-5 gene expression (p*p* < 0.01) with values greater at 5 % oxygen tension than 21 %. The induction of catabolic mediators by TNFα was reduced by dynamic compression and/or L-NIO (all *p* < 0.001), with a greater inhibition observed at 5% than 21 %. The stimulation of GAG synthesis by dynamic compression was greater at 21 % than 5 % oxygen tension and this response was reduced with TNFα or reversed with L-NIO.

**Conclusions:**

The present findings revealed that TNFα increased production of NO, PGE_2_ and MMP activity at 5 % oxygen tension. The effects induced by TNFα were reduced by dynamic compression and/or the NOS inhibitor, linking both types of stimuli to reparative activities. Future therapeutics should develop oxygen-sensitive antagonists which are directed to interfering with the TNFα-induced pathways.

## Introduction

Osteoarthritis (OA) is a progressive joint disease typically characterized by cartilage loss, joint pain and dysfunction. In OA, chondrocytes produce greater levels of pro-inflammatory cytokines such as interleukin 1β (IL-1β) and tumour necrosis factor alpha (TNFα) which increase breakdown of the extracellular matrix [1–3]. Whilst IL-1β is the principal cytokine known to drive breakdown of cartilage, TNFα will also contribute to the pro-inflammatory process in chondrocytes [4–7]. There is also strong evidence that biomechanical signals and oxygen tension will additionally cross-talk with the pro-inflammatory process and activate changes that influence tissue remodelling [8–13]. However, little is known about the combined effects of oxygen tension and TNFα on triggering the pro-inflammatory events and how the pathways are regulated by biomechanical signals.

Several in vitro studies have demonstrated that treatment of chondrocytes with TNFα increases production of nitric oxide (NO), prostaglandin E_2_ (PGE_2_), matrix metalloproteinase (MMP)-1, 3 and 13 and a disintegrin and metalloproteinase with thrombospondin motifs (ADAMTS-5) in human, rabbit, canine or bovine chondrocytes cultured in monolayer, explant or 3D alginate models [5–7, 14–22]. The effects induced by TNFα were shown to be mediated by the mitogen activated protein kinase (MAPK) family or nuclear factor-kappa B (NFκβ), leading to increased proteoglycan depletion and expression of MMP-1, 3 and 13, NO and PGE_2_ [23–26]. The NFκβ transcription factor was reported to control the expression of several cytokines or MMP enzymes [27, 28] and its regulation by oxygen tension will influence the catabolic effects of the cytokine [29–33]. In addition, oxygen tension was reported to influence the production of NO and PGE_2_ in response to biomechanical signals. For example in the chondrocyte/agarose model, low oxygen tension at 5 % enhanced the production of NO and PGE_2_ release in constructs cultured with IL-1β when compared to 21 % oxygen tension and this response was abolished by dynamic compression [34]. However, the combined effect of TNFα and biomechanical signals on the production of NO and PGE_2_ in chondrocytes at 5 % oxygen tension is not known. The present study therefore examined whether low oxygen tension could influence the response of chondrocytes to TNFα and dynamic compression by comparing markers for catabolic activity (NO, PGE_2_) and tissue remodelling (GAG, MMP-13, ADAMTS-5) at 5 and 21 % oxygen tension.

## Materials and methods

### Chondrocyte isolation and culture in agarose constructs

Full depth slices of articular cartilage were isolated from freshly slaughtered cattle aged <18 months (Dawn Cardington, Bedfordshire, UK), as previously described [35]. Cartilage tissue was pooled from two joints, diced and incubated on rollers for 1 h at 37 °C in Dulbecco’s modified Eagle’s medium supplemented with 20 % (v/v) foetal calf serum (DMEM + 20 % FCS), 2 µM l-glutamine, 5 µg ml penicillin, 5 μg/ml streptomycin, 20 mM Hepes buffer, and 0.85 μM l-ascorbic acid + 700 unit/ml pronase, and for a further 16 h at 37 °C in DMEM + 20 % FCS, supplemented with 100 unit/ml collagenase type XI (Sigma Chemical Co., Poole, UK). The cell suspension was washed and viable chondrocytes counted using a haemocytometer and Trypan blue. Cells were resuspended in medium at a cell concentration of 8 × 10^6^ cells/ml. The cell suspension was added to an equal volume of molten 6 % (w/v) agarose type VII (Sigma Chemical Co., Poole, UK) in Earle’s Balanced Salt Solutions (EBSS, Sigma Chemical Co., Poole, UK) to yield a final cell concentration of 4 × 10^6^ cells/ml in 3 % (w/v) agarose. The chondrocyte/agarose suspension was transferred into a sterile stainless steel mould, containing holes 5 mm in diameter and 5 mm in height and allowed to gel at 4 °C for 10 min to yield cylindrical constructs. All constructs were equilibrated in culture in 1 ml DMEM + 20 % FCS at 5 or 21 % oxygen tension for 72 h.

### Effect of oxygen tension and TNFα in chondrocyte/agarose constructs

The effect of 5 and 21 % oxygen tension were examined in constructs cultured under free-swelling conditions in a glove-box style workspace integrated within a Biospherix incubator to ensure that the experimental conditions during setup and experimentation were uninterrupted, as previously described [34]. Constructs were cultured with TNFα (Peprotech EC Ltd, London, UK) at concentrations ranging from 0.1, 1, 10 and 100 ng/ml in the presence and absence of 1 mM l-*N*-(1-iminoethyl)-ornithine (L-NIO) for up to 48 h. L-NIO inhibits all isoforms of the nitric oxide synthase enzymes (Merck Chemicals, Nottingham, UK).

### Application of dynamic compression

In separate experiments, a novel ex vivo bioreactor (Bose ElectroForce, Gillingham, UK) was used to apply dynamic compression to constructs cultured at 5 or 21 % oxygen tension, as previously described [34]. Constructs were transferred into individual wells of a 24-well culture plate (Costar, High Wycombe, UK) and mounted within the bioreactor system that is integrated within the Biospherix incubator. The medium was supplemented with either 0 or 10 ng/ml TNFα in the presence and absence of 1 mM L-NIO and the experimental conditions during setup and culture were uninterrupted. Constructs were subjected to intermittent compression under unconfined conditions, with a profile of 10 min compression followed by a 5 h 50 min unstrained period for both the 6 and 48 h culture periods, as previously described [34]. The ex vivo conditions were found to be optimal when measuring gene expression and protein synthesis at these time points [34]. The compression regime was applied in a dynamic manner with a strain amplitude of 0–15 % using a sinusoidal waveform at a frequency of 1 Hz and resulted in duty cycles which ranged from 600 to 4800 cycles for the 6 and 48 h culture periods, respectively. Control constructs were maintained in an unstrained state in the bioreactor system and cultured for the same time period. At the end of the experiment, the constructs and corresponding media were stored at −70 °C prior to analysis.

### Biochemical analysis

NO and PGE_2_ production were determined in supernatant by Griess and EIA (GE Healthcare, Buckinghamshire, UK) using well established methods [34, 35]. Total MMP activity was measured in media samples using a fluorogenic substrate assay. 25 µl sample was incubated with 2.5 µM amino-phenyl mercuric acetate (APMA) for 1 h at room temperature to activate latent MMPs. Each sample was subsequently mixed with an equal volume of 10 µM Dnp-PChaGCHAK(Nma) fluorogenic MMP substrate, 50 µl buffer (500 mM HEPES, 100 mM CaCl_2_, 0.5 % Brij-35, pH 7.0) in a 96-well plate (Enzo Life Sciences, Exeter, UK) and reactions measured at excitation and emission values of 340 and 440 nm, respectively. The change in fluorescence was calculated in the linear region of the kinetic assay for each sample between a period of 5 and 60 min at 37 °C. GAG synthesis was measured in constructs and media samples by DMMB assay and normalized to DNA values measured using the Hoescht dye 33258 in agarase/papain digests, as described [34, 35].

### RNA extraction, cDNA synthesis and real-time quantitative PCR

RNA was isolated from chondrocytes cultured in agarose as previously described (Qiagen, West Sussex, UK) [34, 36]. RNA was quantified on the Nanodrop ND-1000 spectrophotometer (LabTech, East Sussex, UK) and reverse transcription was performed using the Enhanced Avian RT First Strand cDNA synthesis kit, oligo(dT)_23_ primer, and a total of 200 ng of RNA (Sigma Genosys, Cambridge, UK) according to the manufacturer’s protocols. Real-time quantitative PCR were performed in 10 µl reaction mixtures containing 2.5 µl cDNA, 5 µl Kapa SYBR^®^ FAST Universal qPCR Master Mix (2X) (Kapa Biosystems, Wilmington, Massachusetts, USA), primer pairs at final concentrations of 0.5 μM and nuclease-free PCR-grade water to 10 µl (Sigma Genosys, Cambridge, UK). The following specific primer sequences were used: ADAMTS-5 (NM_001166515) Forward: 5′-GCCCTGCCCAGCTAACGGTA-3′, Reverse: 5′-CCCCCGGACACACACGGAA-3′, MMP-13 (NM_174389.2) Forward: 5′-CCCTTGATGCCATAACCAGT-3′, Reverse 5′-GCCCAAAATTTTCTGCCTCT-3′ and 18S (NR_036642.1) Forward: 5′-GCAATTATTCCCCATGAACG-3′, Reverse: 5′-GGCCTCACTAAACCATCCAA-3′. Each sample was run in duplicate on the 96-well thermal system of the Mx3000P quantitative PCR instrument (Stratagene, Amsterdam, The Netherlands). Thermocycling conditions comprised an initial polymerase activation step at 95 °C for 3 min, followed by denaturation of 35 cycles at 95 °C for 30 s, annealing at 55 °C for 1 min, and extension at 72 °C for 1 min. Fluorescence data were collected during the annealing stage of amplification, and data were analysed on the MxPro qPCR software (version 3, Stratagene). Baselines and thresholds were automatically set by the RG-3000 qPCR software and used after manual inspection. The cycle threshold (Ct) value for each duplicate reaction was expressed as the mean value, and the results were exported into Microsoft Excel for further analysis. The data obtained by PCR assay for 18S were validated as a reference gene by displaying the Ct values as box-and-whisker plots, and the distribution examined under the treatment conditions (data not shown). The Ct values for 18S remained stable, with no changes detected under all culture conditions, suggesting its suitability as a reference gene. Relative quantifications of ADAMTS-5 and MMP-13 signals were estimated by normalizing each target to the reference gene, 18S, and to the calibrator sample by a comparative Ct approach. For each sample, the ratio of target ∆Ct and reference ∆Ct was calculated, as previously described [36, 37]. Ratios were expressed on a logarithmic scale (arbitrary units).

### Statistics

For free-swelling studies, data represent the mean and SEM values for 6–18 replicates from 3 to 4 separate experiments. For the ex vivo bioreactor experiments, data represent the mean and SEM values of 8–12 replicates from two separate experiments. Statistical analysis was performed by a two-way analysis of variance (ANOVA) and the multiple post hoc Bonferroni-corrected *t* tests to compare differences between the various treatment groups as indicated in the figure legend. In all cases, a level of 5 % was considered statistically significant (*p* < 0.05).

## Results

### Low oxygen tension dose-dependently increased production of catabolic mediators in chondrocytes treated with TNFα

**Fig. 1:**
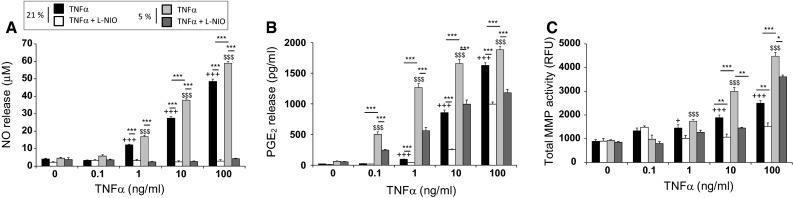
Effect of 5 and 21 % oxygen tension in chondrocytes treated with varying concentrations of TNFα. Chondrocyte/agarose constructs were cultured for 48 h with varying concentrations of TNFα (0.1–100 ng/ml) and/or L-NIO (1 mM) and the effects of 5 and 21 % oxygen tension were examined on NO release (**a**), PGE_2_ release (**b**) and total MMP activity (**c**). *Error bars* represent the mean and SEM values for 6–18 replicates from four separate experiments. *+* or *+++* indicates significant comparisons between untreated and cytokine-treated constructs cultured at 21 % oxygen tension; $ or $$$ indicates significant comparisons between untreated and cytokine-treated constructs cultured at 5 % oxygen tension; ** or *** indicates significant comparisons between TNFα and TNFα + L-NIO

At 5 and 21 % oxygen tensions, the levels of NO were enhanced by TNFα, with a significant increase at 1, 10 and 100 ng/ml, when compared to untreated controls (all *p* < 0.001; Fig. 1a). The NOS inhibitor abolished cytokine-induced NO release with levels returning to basal values at both 5 and 21 % oxygen. At 10 and 100 ng/ml TNFα, the levels of NO release were greater at 5 % oxygen tension when compared to 21 % (both *p* < 0.001). TNFα dose-dependently increased PGE_2_ release with values greater at 5 % than 21 % oxygen tension (all *p* < 0.001; Fig. 1b). These effects were partially reversed with the NOS inhibitor (all *p* < 0.001). TNFα concentrations greater than 1 ng/ml increased MMP activity in a dose-dependent manner at both oxygen tension when compared to untreated controls (Fig. 1c). Co-incubation with the NOS inhibitor partially reversed this effect in TNFα-treated constructs cultured at either 5 or 21 % oxygen. At 10 and 100 ng/ml, MMP activity was significantly greater at 5 % oxygen when compared to 21 % (all *p* < 0.001).

### Low oxygen tension dose-dependently influenced matrix synthesis and loss in chondrocytes treated with TNFα

**Fig. 2:**
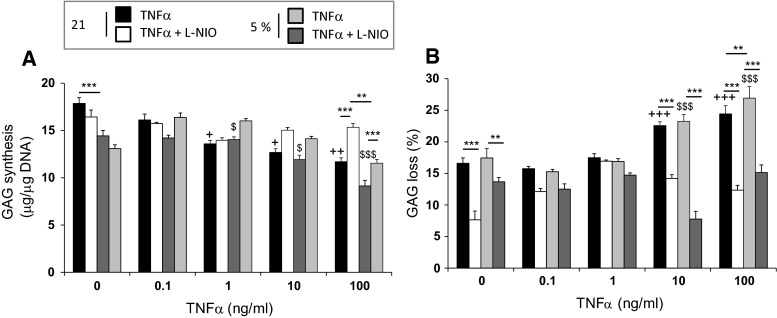
Dose-dependent effects of TNFα on matrix synthesis and degradation at 5 and 21 % oxygen tension. Chondrocyte/agarose constructs were cultured with varying concentrations of TNFα (0.1–100 ng/ml) and/or L-NIO (1 mM) for 48 h and the effects of 5 and 21 % oxygen tension were examined on GAG synthesis (**a**) and GAG loss (**b**). *Error bars* represent the mean and SEM values for 6–18 replicates from four separate experiments. *+* or +++ indicates significant comparisons between untreated and cytokine treated constructs cultured at 21 % oxygen tension; $ or $$$ indicates significant comparisons between untreated and cytokine-treated constructs cultured at 5 % oxygen tension; ** or *** indicates significant comparisons between TNFα and TNFα + L-NIO

In the absence of the cytokine, GAG synthesis was greater at 21 % oxygen when compared to 5 % (*p* < 0.001; Fig. 2a). At low cytokine concentration (0.1 ng/ml), TNFα did not significantly influence GAG synthesis when compared to untreated controls cultured at either 5 or 21 % oxygen (Fig. 2a). At TNFα concentrations of 1–100 ng/ml, the cytokine downregulated GAG synthesis (*p* < 0.05) but the response was not influenced by the NOS inhibitor, except at 100 ng/ml of TNFα. At the highest cytokine concentration (100 ng/ml), the inhibitory effect on GAG synthesis was greater at 5 % oxygen when compared to 21 % (*p* < 0.01). In the absence of the cytokine, GAG loss was inhibited with L-NIO at both 5 and 21 % oxygen (*p* < 0.01 and *p* < 0.001, respectively; Fig. 2b). At TNFα concentrations of 10–100 ng/ml, the cytokine increased GAG loss (*p* < 0.05), and this response was reversed with the NOS inhibitor. At 100 ng/ml TNFα, the increase in GAG loss was greater at 5 % oxygen when compared to 21 % (*p* < 0.01).

### Dynamic compression reduced TNFα induced catabolic effects at 5 and 21 % oxygen

**Fig. 3:**
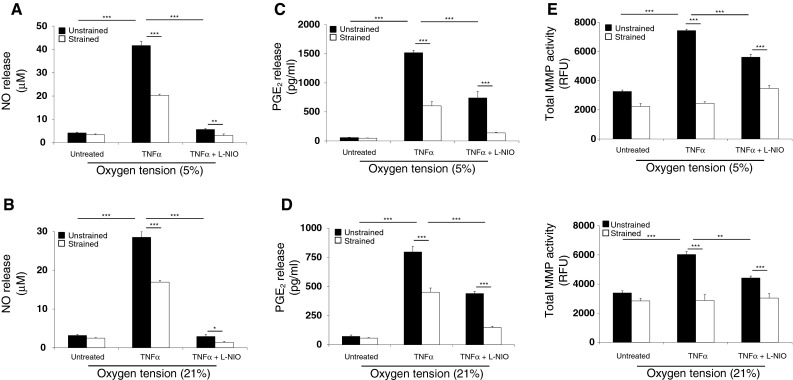
The effects of TNFα and dynamic compression on the production of catabolic mediators at 5 and 21 % oxygen tension. Chondrocyte/agarose constructs were subjected to dynamic compression (15 %, 1 Hz) in the presence or absence of TNFα (0 or 10 ng/ml) and/or L-NIO (1 mM) for 48 h and the effects of 5 and 21 % oxygen were examined on NO release (**a**, **b**), PGE_2_ release (**c**, **d**) and total MMP activity (**e**, **f**). *Error bars* represent the mean and SEM values for 8–12 replicates from four separate experiments. *, ** or *** indicates significant comparisons between the different treatment conditions. All other comparisons were not significant (not indicated)

Figure 3 reveals that in the absence of the cytokine, dynamic compression did not significantly influence NO release at either 5 or 21 % oxygen tension. In unstrained constructs, TNFα enhanced NO production with a greater effect at 5 % oxygen (41.6 µM) when compared to 21 % oxygen (28.5 µM), and the response was reduced with dynamic compression (all *p* < 0.001; Fig. 3a, b). The magnitude of inhibition was greater at 5 than 21 % oxygen, as indicated in Table 1, and the inhibitory effect abolished with L-NIO (*p* < 0.001).

**Table 1:** The effects of oxygen tension and dynamic compression on catabolic/remodelling activities in chondrocyte/agarose constructs treated with TNFα

	NO release		PGE_2_ release		MMP activity		GAG synthesis	
	21 %	5 %	21 %	5 %	21 %	5 %	21 %	5 %
Untreated	−20.6 (±4.4)	−19.9 (±8.3)	−20.8 (±4.7)	−17.0 (±7.6)	−16.1 (±4.9)	−34.4 (±7.3)	47.9 (±3.1)	32.9 (±5.5)
TNFα	−39.9 (±2.1)	−50.7 (±2.1)	−43.8 (±3.3)	−60.3 (±4.8)	−43.8 (±6.6)	−67.1 (±5.1)	106.7 (±24.2)	82.3 (±7.2)
TNFα + L-NIO	−45.3 (±8.9)	−47.6 (±14.6)	−66.2 (±2.6)	−79.9 (±2.5)	−66.2 (±7.3)	−38.1 (±5.9)	64.4 (±2.9)	90.9 (±5.9)

Chondrocyte/agarose constructs were subjected to dynamic compression in the presence and absence of TNFα and/or L-NIO at 5 and 21 % oxygen tension for 48 h. Values were expressed as a percentage change from unstrained control samples (%) where numbers in brackets represent **±**SEM values for *n* = 8–12 from four separate experiments

In the absence of the cytokine, dynamic compression did not significantly influence PGE_2_ release at either oxygen tension (Fig. 3c, d). In unstrained constructs, TNFα increased PGE_2_ release when compared to untreated controls (*p* < 0.001), with values greater at 5 % (1517.5 pg/ml) than 21 % (795.1 pg/ml) oxygen tension. Stimulation with either dynamic compression or the NOS inhibitor reduced cytokine-induced PGE_2_ release (all *p* < 0.001) with the magnitude of inhibition greater at 5 % than 21 % oxygen tension (Table 1). Co-stimulation with both compression and the NOS inhibitor induced a further reduction in PGE_2_ release (all p < 0.001) with the magnitude of inhibition greater at 5 % than 21 % (Table 1).

In the absence of the cytokine, dynamic compression did not significantly influence MMP activity at either oxygen tension (Fig. 3e, f). In unstrained constructs, the cytokine increased MMP activity when compared to untreated controls (*p* < 0.001), with values marginally greater at 5 % than 21 % oxygen (*p* < 0.001). Stimulation with dynamic compression or the NOS inhibitor reduced cytokine-induced MMP activity (all *p* < 0.001) with the magnitude of inhibition greater at 5 % than 21 % oxygen (Table 1). In the presence of TNFα, co-stimulation with compression and L-NIO reduced MMP activity (all *p* < 0.001) with the magnitude of inhibition broadly similar at both oxygen tensions (Table 1).

### Dynamic compression reversed TNFα-induced GAG synthesis at 5 and 21 % oxygen

**Fig. 4:**
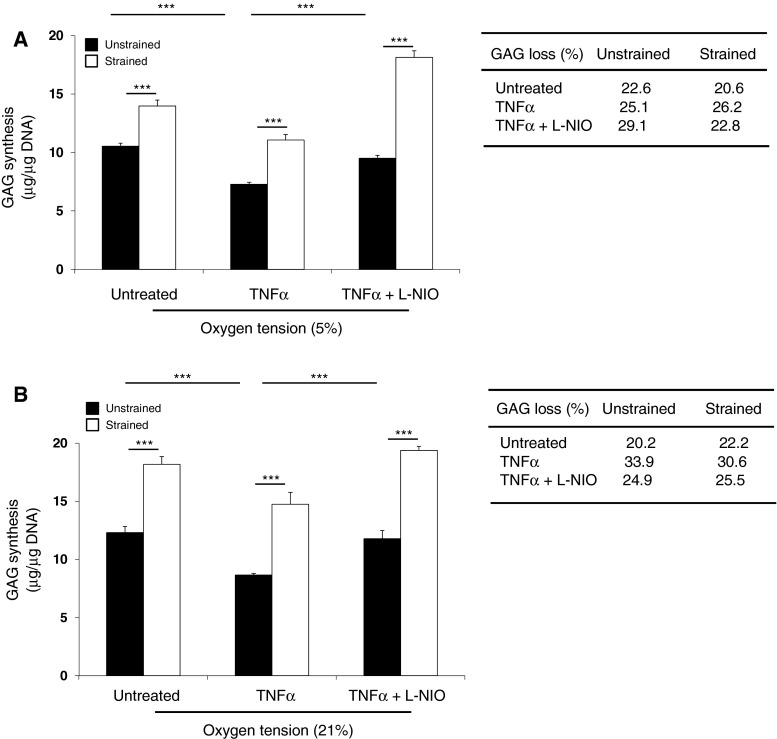
The effects of TNFα and dynamic compression on GAG synthesis and loss in constructs cultured at 5 and 21 % oxygen. Chondrocyte/agarose constructs were subjected to dynamic compression (15 %, 1 Hz) in the presence or absence of TNFα (0 or 10 ng/ml) and/or L-NIO (1 mM) at 5 and 21 % oxygen tension for 48 h where GAG synthesis (**a**, **b**) and GAG loss (*inset*). *Error bars* represent the mean and SEM values for 8 replicates from two separate experiments. *** indicates significant comparisons between the different treatment conditions. All other comparisons were not significant (not indicated)

GAG synthesis was enhanced by dynamic compression when compared to unstrained controls (all *p* < 0.001; Fig. 4) with a greater magnitude in stimulation for constructs cultured at 21 % oxygen than 5 % (Table 1). At both oxygen tensions, TNFα reduced GAG synthesis (all *p* < 0.01; Fig. 4a, b). Dynamic compression increased GAG synthesis in cytokine-treated constructs (both *p* < 0.001), with the magnitude of stimulation greater at 21 % oxygen than 5 %. Co-stimulation with dynamic compression and L-NIO increased GAG synthesis (*p* < 0.001, Fig. 4a, b) with the magnitude of stimulation by dynamic compression greater at 5 % oxygen than 21 % (90.9 vs 64.5 %, respectively). GAG loss was not influenced by compression at either oxygen tension (Fig. 4a, b, inset).

### Low oxygen tension increased expression of MMP-13 and ADAMTS-5 in chondrocytes treated with TNFα and the response was reduced by dynamic compression

**Fig. 5:**
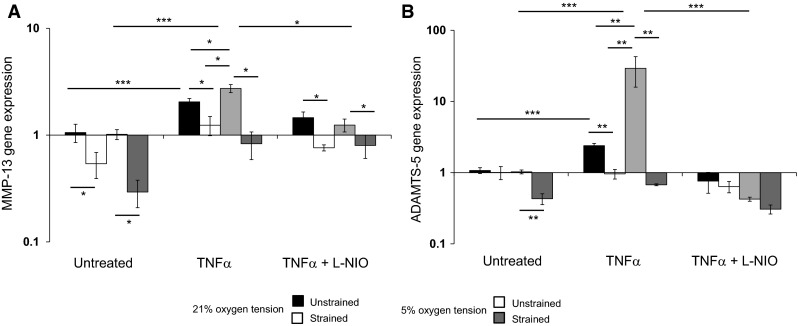
The effects of TNFα and dynamic compression on MMP-13 and ADAMTS-5 gene expression at 5 and 21 % oxygen. Chondrocyte/agarose constructs were subjected to dynamic compression (15 %, 1 Hz) in the presence or absence of TNFα (0 or 10 ng/ml) and/or L-NIO (1 mM) at 5 and 21 % oxygen tension for 48 h where MMP-13 (**a**) and ADAMT-S5 (**b**) gene expression. *Error bars* represent the mean and SEM values for 6 replicates from two separate experiments. *** indicates significant comparisons between the different treatment conditions. All other comparisons were not significant (not indicated)

We examined whether oxygen tension influenced gene expression of MMP-13 and ADAMTS-5 in chondrocytes cultured with TNFα and subjected to dynamic compression. At 5 and 21 % oxygen tensions, the presence of TNFα increased gene expression of MMP-13 and ADAMTS-5 when compared to untreated controls (both *p* < 0.001; Fig. 5). In unstrained constructs, we observed greater levels of MMP-13 (*p* < 0.05) and ADAMTS-5 (*p* < 0.01) gene expression at 5 % oxygen tension than 21 % oxygen tension. The induction of MMP-13 and ADAMTS-5 gene expression by TNFα at 5 and 21 % oxygen tension were reduced with the NOS inhibitor or abolished by stimulation with dynamic compression.

## Discussion

TNFα is well known to stimulate production of catabolic mediators such as NO and PGE_2_ which inhibit matrix synthesis and induce cartilage degradation [7, 15–22]. The in vitro studies correlate with previous animal studies which showed that selective inhibition of iNOS reduced the symptoms of inflammation and biomechanical abnormalities in osteoarthritic joints [38–40]. However, the overproduction of cytokines in response to oxygen tension and the effect of biomechanical signals on the cell signalling process is less clear. Indeed, the levels of oxygen tension in the diseased joint will have a significant impact on metabolic processes, with the potential to trigger pathways induced by TNFα. The interactions between cytokines, oxygen tension and mechanical loading are therefore complex and require further investigation.

In ex vivo studies, we observed dose-dependent increases in NO, PGE_2_ and MMPs, that was paralleled with an inhibition of matrix synthesis and loss at the highest cytokine concentration. Reduced oxygen tension at 5 % was observed to enhance the effects induced by TNFα with greater induction of MMP-13 and ADAMTS-5 gene expression and levels of NO, PGE_2_ and MMP activity that also favours the inhibition of matrix synthesis and loss. In a previous study, bovine chondrocytes stimulated with IL-1β in suspension culture exhibited a similar response, with greater levels of NO and PGE_2_ production at 5 % when compared to 21 % oxygen tension [30]. The enhanced production of NO under hypoxic conditions can contribute to the production of reactive oxygen species (ROS) that amplifies the catabolic response [12]. Furthermore, the p55 TNFα receptor is highly expressed in human chondrocytes from OA cartilage and is particularly susceptible to degradative stimuli [41]. Activation of p55 by TNFα was shown to increase synthesis of NO, PGE_2_, MMPs and cytokines such as IL-6, IL-8 that degrade collagen type II, IX and XI and inhibit matrix synthesis in a concentration-dependent manner [20–22, 42–44]. However, studies on the effect of low oxygen tension in chondrocytes have resulted in conflicting outcomes. Porcine explants treated with IL-1α or TNFα increased levels of NO and PGE_2_ under normoxic conditions (21 %) when compared to severe hypoxic conditions (1 %) [31]. In contrast, cytokine-treated chondrocytes induced a reduction in oxidative stress resulting in reduced MMP-9 levels at moderate hypoxia (6 %) when compared to normoxia (21 %) and stablization of hypoxia-inducible factor-1α (HIF-1α) expression [32]. Indeed, the regulation of HIF-1α by oxygen tension may present a potential target for OA therapy, since HIF-1α over-expression in OA chondrocytes is known to have detrimental effects in cartilage pathophysiology. Furthermore, factors involved in the NFκβ and MAPK pathways were shown to mediate production of NO induced by the cytokine at 5 % oxygen tension, presenting supplementary oxygen-sensitive mediators as potential therapeutic targets for treating OA [30, 33]. Collectively, these studies emphasize the oxygen-dependency of the pro-inflammatory induced effects in chondrocytes and suggest that further studies should examine the interplay of the cytokine-induced pathways with oxygen tension.

In ex vivo bioreactor studies, dynamic compression reduced the production of inflammatory mediators in response to TNFα, and this response was abolished when dynamic compression was coupled with the NOS inhibitor. We observed differences in the loading-induced response such that the magnitude of inhibition was greater at 5 % oxygen tension than 21 %. In addition, the beneficial response was paralleled with anabolic activities as typified by increased matrix synthesis, that was greater at 21 % oxygen tension than 5 %. The literature is sparse with respect to the combined effect of TNFα and dynamic compression at low oxygen tensions in chondrocytes. However, the effect of oxygen tension and mechanical stress is well characterized [11, 44, 45]. Matrix synthesis was increased and chondrogenic gene expression was stabilized by long-term mechanical loading at 5 % oxygen tension when compared to 21 % in a chondrocyte/polyurethane model [45]. A similar effect was observed in the alginate model which reported a greater production of GAG synthesis at 5 % oxygen tension compared to 20 % [46]. The differences observed in the present study are due to the type of model system used, e.g. cell type, 2D vs 3D model, primary vs passage cells, free-swelling culture vs mechanical loading, uninterrupted oxygen tension using the biospherix system vs oxygen controlled incubators [47, 48]. Conversely in porcine cartilage explants, mechanical loading enhanced NO production at 5 and 20 % oxygen tension and the response was reduced at 1 % oxygen tension [11, 44]. However, the manner in which cytokine-induced inflammatory pathways are influenced by oxygen tension and biomechanical signals are unclear. Further studies are needed to unravel the distinct pathways induced by oxygen tension, biomechanical signals and TNFα. This will help to identify key targets and potential therapies for OA.

In summary, the present study demonstrates that exogenous TNFα combined with low oxygen tension enhanced the production of NO, PGE_2_ and MMPs. The effects of TNFα were reduced with biomechanical signals or the presence of the NOS inhibitor in an oxygen-dependent manner, leading to restoration of matrix synthesis. Although selective inhibition of NOS and stimulation with biomechanical signals is chondroprotective, further studies are needed to unravel the distinct pathways induced by oxygen tension, biomechanical signals and TNFα. This will help to identify key targets and potential therapies for OA treatments.

## Abbreviations

TNFα: Tumour necrosis factor alpha
NO: Nitric oxide
MMPs: Matrix metalloproteinases
